# The Effectiveness and Sustainability of a Universal School-Based Programme for Preventing Depression in Chinese Adolescents: A Follow-Up Study Using Quasi-Experimental Design

**DOI:** 10.1371/journal.pone.0149854

**Published:** 2016-02-26

**Authors:** Eliza S. Y. Lai, Chi-Leung Kwok, Paul W. C. Wong, King-Wa Fu, Yik-Wa Law, Paul S. F. Yip

**Affiliations:** 1 The Hong Kong Jockey Club Centre for Suicide Research and Prevention, The University of Hong Kong, Hong Kong; 2 Department of Social Work and Social Administration, Faculty of Social Sciences, The University of Hong Kong, Hong Kong; 3 Journalism and Media Studies Centre, The University of Hong Kong, Hong Kong; Istituto Superiore di Sanità, ITALY

## Abstract

**Background:**

A pilot study about the effectiveness of a universal school-based programme, “The Little Prince is Depressed”, for preventing depression in Chinese adolescents in Hong Kong was conducted and reported previously. This study used a larger sample to examine the effectiveness and sustainability of the programme.

**Methods:**

This study used quasi-experimental design. Twelve schools enrolled in “The Little Prince is Depressed” programme either as an intervention or a control condition. The intervention schools carried out the 12-session programme in two phases: the professional-led first phase and the teacher-led second phase. All participants were required to complete a questionnaire at three time points measuring their (1) depressive, anxiety, and stress levels; (2) knowledge of mental health; (3) attitudes towards mental illness; (4) perceived social support; and (5) help-seeking behaviours.

**Results:**

A total of 3,391 students participated in the study. The level of depressive symptoms did not reduce significantly at post-intervention; however, a delayed effect was observed at follow-up assessment for the participants of the teacher-led group in reducing anxiety and stress levels. Also, the knowledge of mental health and attitudes towards mental illness of the intervention-group participants significantly improved at post-test, and the outcomes were maintained at 4 to 5 months after the intervention in both the professional-led and the teacher-led conditions (p<.05). A preference among schoolchildren for whom to seek help from was identified.

**Conclusions:**

The universal depression prevention programme was effective in enhancing knowledge of mental health and promoting a more positive attitude towards mental illness among adolescents in Hong Kong. In particular, the teacher-led group showed better outcomes than the professional-led group in reducing students’ anxiety and stress at follow-up period. The programme can achieve sustainability in schools if teachers are provided with adequate support.

## Introduction

Depressive disorders have been ranked second and first as the cause of disability in developed countries and in developing countries, respectively [1]. Longitudinal studies of community samples of children and adolescents suggest an average age of onset between 11 and 14 years for depression [2]. A recent review study on the epidemiology of mental disorders in children and adolescents found a median 12-month prevalence estimate of 4.0% with a range from 0.2% to 17% for major depression [3]. The prevalence rates of depressive symptoms and depressive disorders in a 12-month time frame among the youth of Hong Kong have been estimated at about 1.7% and 1.3%, respectively [4].

Depression among adolescents is co-morbid with other mental disorders (e.g. anxiety and alcoholism [5]); as with physical disorders (e.g. obesity [6]), depression may continue into adulthood and result in further burdens, such as occupational, financial, and social difficulties [7]. The serious developmental consequences of adolescent depression underscore the need for prevention programmes [8–10]. Randomized controlled trials have demonstrated that effective psychological interventions are available for the treatment of depression in adolescents, at least in the short term [11–12]; however, the majority of adolescents with depression or depressive symptoms remain unidentified, not to mention untreated [13]. Schools offer a convenient location for the widespread delivery of depression prevention programmes; however, according to a review conducted by Spence and Shrott, widespread implementation of school-based universal interventions for the prevention of depression in children and adolescents is difficult to justify until the interventions are found both efficacious and effective [14]. They suggested that future research designs should be more rigorous; the content of intervention should also include the social environment of the children and adolescents (family, school context, etc.); and training and supervision of those who deliver the intervention should be improved. One intervention programme did attempt to adopt these suggestions: according to a 3-year randomized controlled study on a universal school-based intervention for preventing adolescent depression in Australia [15], the programme was designed with the latest evidence, training was provided for the schoolteachers, and the programme adopted a whole-school approach when being implemented. Nevertheless, there were no statistically significant differences between intervention-group and control-group students regarding depressive symptoms or the levels of risk and protective factors. It therefore remains unclear how to effectively implement a universal intervention programme aimed at preventing depression in adolescents. Another systematic review found that universal school-based prevention and early intervention programmes for depression achieved a lower effect size than indicated and selective programmes [11]. Yet some universal programmes have been found effective and it is not reasonable to compare the effect sizes of interventions with different target populations.

In 2005, we developed and piloted a two-phase universal school-based programme, the “Little Prince is Depressed” (LPD), aimed at preventing adolescent depression in Hong Kong. The programme was initiated because Hong Kong has a relatively small number of psychologists and psychiatrists available to identify adolescents with depression and to provide them with effective treatment; in addition, public understanding of mental health is inadequate. Such considerations prompted us to launch our programme at four secondary schools in Hong Kong in 2006. However, most likely owing to the small number of students participating in the pilot study, the depressive symptoms of the students showed no changes over the period of the study, although students did show positive development in help-seeking attitudes and in self-esteem at post-test. Also, the cognitive-restructuring skills and support-seeking behaviours of students with more depressive symptoms at pre-assessment were enhanced [16].

This study has extended the pilot study: we revised the programme based on the process and outcome evaluation findings in the first phase, and it was implemented in a larger number of schools, thus it covered a larger sample. We examined the effectiveness of the programme in reducing depressive symptoms and explored the sustainability of such school-based prevention programmes by examining the changes in outcomes between classes led either by mental-health professionals or teachers. We reasoned that if teachers could be as effective as mental-health professionals in implementing programmes of this sort, there would be a much higher chance for such programmes to be sustained at the participating schools, especially when funding for such programmes was not available; though previous similar studies seemed to find stronger effects when programmes were implemented by mental-health professionals but much weaker effects when they were implemented by teachers [17]. The Wahl et al. study [17] had reviewed the literature about the use of psychologists or teachers to be the instructors of school-based prevention programmes for depression and highlighted the need to have direct comparison of the effects between psychologist-led and teacher-led programme for preventing depression. More evidence about the effectiveness of different kinds of instructors would help stakeholders to decide appropriate strategies for implementing school mental health programmes. To the best of our knowledge, probably the current study is the first of its kind to directly compare the effects of mental health professionals with school teachers in implementing a universal, school-based depression prevention programme in Hong Kong; it could therefore extend our existing knowledge on sustainability of school-based mental health programmes in non-Western cultures. In addition, the current study not only measured the depressive symptoms of programme participants but also other constructs related to depression and help-seeking that were important for preventing depression in adolescents.

In this study, symptoms of depression, anxiety and stress were the primary outcomes, whereas knowledge of mental health, perceived social support, attitudes towards people with mental illness and help-seeking were the secondary outcomes. As the LPD programme was a prevention programme for depression, the symptoms of depression and other emotional symptoms that were related to depression, such as anxiety and stress, were measured to detect the changes of students before and after the programme. For the secondary outcomes, knowledge of mental health—known as “mental health literacy”—was included because good mental health literacy may lead to early identification and intervention of mental health problems, help-seeking attitude and behaviours by adolescents as well as helping adults [18]. “Mental health literacy” is defined as “knowledge and beliefs about mental disorders which aid their recognition, management or prevention” [19]. In the LPD programme, causes, symptoms and treatment of depression, as well as differences between emotional distress and depression, were taught, and the importance of help-seeking and whom to seek help from were highlighted. In addition, the skills taught in the programme also formed part of the knowledge checklist so that the level of knowledge obtained from the programme could be measured. The skills were developed based on Cognitive-Behavioural therapy, such as the identification of thinking errors, cognitive restructuring skills, communication skills and problem-solving skills.

Another secondary outcome, perceived social support, was included because social support was regarded as a protective factor of depression based on existing literature. It can be a buffer for the potential adverse effects of stressful events or may produce direct helpful effects on an individual [20]. Studies showed that there was higher prevalence of depression for those who lack social support [21] and perceived parental and peer support could buffer against adolescent depression [22]. By teaching students communication skills and conflict resolution in the LPD programme, it was hoping that students could improve their interpersonal relationship after acquiring the skills and enhance their perceived social support, which might protect them from depression.

Attitudes toward mental illness were included as another secondary outcome because one’s attitude might affect whether one will seek help or encourage other people to seek help. Studies showed that when there is public or self-stigma on mental illness, people might avoid receiving treatment because they do not want to be labelled as mentally ill [23]. By teaching the knowledge of mental health and mental health problems in the LPD programme, it was hoped that attitudes towards mental illness could be improved with more understanding of people with mental illness.

The last secondary outcome was attitude towards help seeking, and in particular from whom would people seek help. Reviews showed that the most common barrier for adolescents or young adults to seek help for mental health problems was stigma of mental illness—either it was public, perceived or self-stigma [24–25]. Some other proposed reasons for not seeking help included relying on oneself to solve emotional problems, lack of mental health literacy to identify symptoms and helpful resources, and lack of emotional competence to express their feelings. Even though adolescents were going to seek help when they were emotionally distressed, it was more common for them to seek help or recommend someone to seek help from counsellors, family or friends instead of professional like psychologist or psychiatrist [24, 26]. To facilitate adolescents to seek help, it was suggested that enhancing their mental health literacy and emotional competence, and having social support or encouragement from others, might do [24–25]. Therefore, in addition to the knowledge of mental health, perceived social support and attitude towards mental illness, attitude towards help seeking was measured in order to understand whether students receiving the LPD programme would have changes to their help-seeking attitude when under emotional distress, especially towards professional help.

In this study, we hypothesized that, compared with adolescents who did not receive the programme (i.e. the control group), those who participated in the universal prevention programme would (1) report significantly lower levels of depressive symptoms; (2) report significantly higher levels of knowledge and perceived social support, develop more positive attitudes towards people with mental illness, and help-seeking; and (3) both professional-led and teacher-led classes might produce similar patterns of results.

## Method

### Participants

The participants were 3,391 Secondary 2 to 4 students (equivalent to Grades 8–10) from 12 secondary schools (nine co-educational, one girls’ and two boys’ schools). They were mostly aged 14 to 16 (91%).

### Procedures

The 12-session school-based programme was carried out in 2009–2011. Two information seminars about the programme were organized in November 2008 and February 2010 for recruitment of schools. All 498 local secondary schools were invited to attend the seminars. Representatives from 20 schools and 59 schools joined the first and the second seminar, respectively. After the first seminar, five schools joined the programme as the intervention group and three schools agreed to be the control group. The three control schools actually showed interest to join the programme at the time of enrollment. However, after detailed discussion, they declined to participate due to mismatch between programme and school schedules. Therefore, the research team invited them to be the control schools and they agreed to do so. After the second seminar, four more schools joined the programme; two of them acted as intervention group while another two had both intervention and control groups: one grade of students was the intervention group and another grade in the same school was the control group. This arrangement was adopted to ensure an adequate number of students were in the control group, as the research team could not find control schools at the beginning of the second school year (i.e. 2010–11). Two months after the second school year had begun, we invited two control schools recruited from the first information seminar to be the control group again (a new group of students different from the first school year). Thus, altogether seven schools were in the intervention group, three schools acted as the control group, and two schools acted as both intervention and control groups.

The LPD programme primarily targeted Secondary 2 and Secondary 3 students (equivalent to Grade 8 and 9 in US system respectively). Randomization at class level was not preferable by the participating schools because they preferred all students of the same grade to either receive or not receive the programme. They chose the grade of students to receive the programme based on the need of their students and operational concern. For example, whether the curriculum was suitable for particular grade of students and whether they could arrange adequate lessons to conduct the programme. The lack of random assignment made the study a quasi-experimental design.

In order to obtain a control group as comparative as possible, the research team decided which grade of students to join the research study in the three control schools. For example, as exactly one boys’ school existed in both intervention group and control group, we invited the same grade of students from the control school according to the decision of that intervention school (i.e. Secondary 3). For the two schools with both intervention and control groups, invitations to also join the control group were issued after they had already decided which grade of students to join the programme as intervention group. This was an ad-hoc arrangement in order to increase the sample size of the control group, as explained above. In particular, one school had their Secondary 2 students participating in the programme and their Secondary 3 students as control group, while another school had the opposite decision. Ultimately, we maintained similar ratios of Secondary 2 to Secondary 3 students between intervention group and control group.

There were two phases in the study. Phase one was called the “professional-led phase” and involved trained postgraduates of counselling or social work teaching the LPD programme in the first year (i.e. the school year 2009–10) while schoolteachers observed in the classroom and received additional 3 hours of training to teach the programme provided by the research team. The postgraduate counsellors and social workers received 12 hours of training by the in-house Clinical Psychologist who had developed the programme materials and a social worker who had conducted the programme in the pilot project a year before the current programme. The training included the knowledge and activities to be delivered in the 12 sessions, the skills to interact with students and the proper attitudes to work with schoolteachers. Phase two was called the “teacher-led phase” and involved trained schoolteachers teaching the LPD programme to their students in the second year (i.e. the school year 2010–11) while the research team provided on-going consultation. The research team also paid school visits during the teacher-led phase to monitor the teaching quality.

The LPD programme consisted of twelve 45- to 60-minute sessions, which took place during regular school hours. The sessions were based on a cognitive-behavioural approach, and the topics were the same as reported in the pilot study [16]; the major difference was that more audio-visual materials (e.g. short videos, animations) were developed to demonstrate the concepts being taught, such as identifying thinking errors, effective communication skills, and problem-solving skills. The teacher and student manuals used in the programme can be found at http://csrp.hku.hk/school.

The study was approved by the Human Research Ethics Committee for Non-Clinical Faculties (HRECNCF) at The University of Hong Kong (reference no. EA160709). In both phases, the school principals of the participating schools gave their written consent to participate in the research study. Any student could opt out from the study at any time. Parents could also opt to withdraw their children from the study. All participants filled in a questionnaire at three time points: 1 to 4 weeks before the first session of the programme (pre-test), within 2 weeks after the last session of the programme (post-test), and 4 to 5 months after the last session (follow-up test). The control group participants were invited to fill in the same questionnaire in a similar time frame to that of the intervention group participants. Parental consent for study participation was sought prior to the pre-test in the form of passive consent, which means parents are fully informed of the right to refuse participation by their children and be given reasonable time to object to the child’s participation. According to the operational guidelines and procedures of HRECNCF, passive parental consent is normally sufficient for school-based research involving secondary school students (aged 11 or above) and minimal risk. HRECNCF had approved this consent procedure. Participant information was collected anonymously, with class number and date of birth used as identifiers for the post-test. Like previous studies [27–28], only data from students who completed both assessments were included in the analyses.

### Measures

#### Primary outcomes

The Depression Anxiety Stress Scale (Short form):

A short version of the Depression Anxiety Stress Scale (DASS21) was used in this study; it consists of 21 items (seven items for each of the three subscales, namely Depression, Anxiety, and Stress). The depression subscale measures low positive affect, the anxiety subscale measures physiological hyper-arousal features that are unique to anxiety, and the stress subscale measures a negative emotional syndrome distinct from general negative affect and non-specific symptoms of depression and anxiety [29]. The reliability—as indicated using Cronbach’s alpha for the depression, anxiety, and stress subscales—was found to be high: 0.88 (95% CI: 0.87–0.89), 0.82 (95% CI: 0.80–0.83), and 0.93 (95% CI: 0.93–0.94) respectively [30]. The factor structure of the Chinese version of DASS, tested among Chinese-speaking Hong Kong adolescents with confirmatory factor analysis, demonstrated that negative emotional syndromes of depression, anxiety, and stress were significantly discriminated and applicable for clinical and non-clinical samples [29]. Participants were asked to rate the extent to which they experienced each item in the past week using a 4-point combined severity/frequency scale that ranged from 0 (“did not apply to me at all”) to 3 (“applied to me very much or most of the time”). The scores obtained were summed and multiplied by a factor of two for the purpose of comparison with the full version of DASS. The scores ranged from 0 to 42 for each subscale, with higher scores indicating higher severity. In the current study, the reliability of all three subscales was high, with Cronbach’s alpha coefficients of 0.88 for depression, 0.84 for anxiety, and 0.86 for stress.

#### Secondary outcomes

Knowledge checklist:

A six-item knowledge checklist was constructed by the research team, aimed at assessing the knowledge of the students before and after the programme. Each item was a multiple-choice question related to the topics of the 12-session programme. Sample items are, “Which of the following is not a symptom of depression? A. Withdrawal from meeting people B. Loss of interest in pleasurable activities C. Auditory hallucination D. Suicidal thoughts”, and “Which of the following is not a thinking error? A. Over-generalization B. Imagining the worst C. Maximize the negatives and minimize the positives D. Blaming oneself or the world.”

Attitudes towards people with mental illness:

Six items about respondents’ attitudes towards people with mental illness were selected from a study conducted by Mehta et al. [31]. Participants were required to indicate on a 5-point Likert scale how much they agree with each item (from -2 “disagree”, -1 “partially disagree”, 0 “neutral”, 1 “partially agree” to 2 “agree”). Sample items are, “There is something about people with mental illness that makes it easy to tell them from normal people”, and “Mental illness is an illness like any other”.

The Multidimensional Scale of Perceived Social Support:

The Chinese version of the Multidimensional Scale of Perceived Social Support (MSPSS) as validated by Chou (α = .89) [32], was used to measure the adequacy of social support assessed subjectively by an individual [33]. The scale has 12 items measured on a 7-point scale (ranging from 1 = strongly disagree to 7 = strongly agree), with higher scores (total scores ranging from 12 to 84) indicating higher levels of perceived social support [32]. The Cronbach’s alpha for family subscale and friend subscale was 0.94 and 0.96 respectively in the current study, indicating excellent internal consistency.

Help-seeking attitude when under emotional distress:

A self-constructed item was used to ask for the respondent’s help-seeking attitude when experiencing emotional distress—whether they will seek professional help, or talk to family, friends, or teachers. Respondents indicated on a 4-point Likert scale (ranging from 1 “disagree” to 4 “agree”) to what extent they will seek help from professionals, family, friends, and teachers.

### Statistical Analysis

Multilevel modelling [34–35] was used to analyse the data. This analytical approach was also employed in an evaluation study of a school-based prevention programme of depression [15]. A three-level regression model was first employed to test if there existed significant group differences (professional-led, teacher-led, and control) during pre-test assessment on each outcome measure, taking into account the clustering of students both within classes and within schools. The differences due to the effects of different subgroups—gender, age and a binary variable of programme starting time (September or October or other)–were included as confounding variables.

To test if intervention effects existed, a series of four-level models was employed for each outcome measure separately. Level 1 accounted for the changes within students on the outcome measure, while levels 2, 3 and 4 accounted for the between-student, between-class and between-school differences respectively. Independent variables included the time variable (pre-test [T0] as baseline, with T1 indicating post-test and T2 indicating follow-up), the group variable, and their interaction term (i.e. time*group). The interaction effect T1*group examined the differences across three groups immediately after completion of the programme. A significant coefficient indicated that the score varied across groups, suggesting the existence of an intervention effect. By the same token, the interaction effect T2*group examined the differences across groups during follow-up assessment, and was used to test if the intervention effect could be found 4 to 5 months after the completion of the programme. Given that the intervention effect was found during post-test assessment, a significant coefficient of interaction effect T2*group indicated the persistence of an intervention effect. If the intervention effect was statistically significant on DASS21, the clinical significance would then be assessed using Jacobson-Truax method [36].

All analyses were performed by PROC MIXED in the statistical programme SAS version 9.3 for Windows [37].

## Results

**Fig 1:**
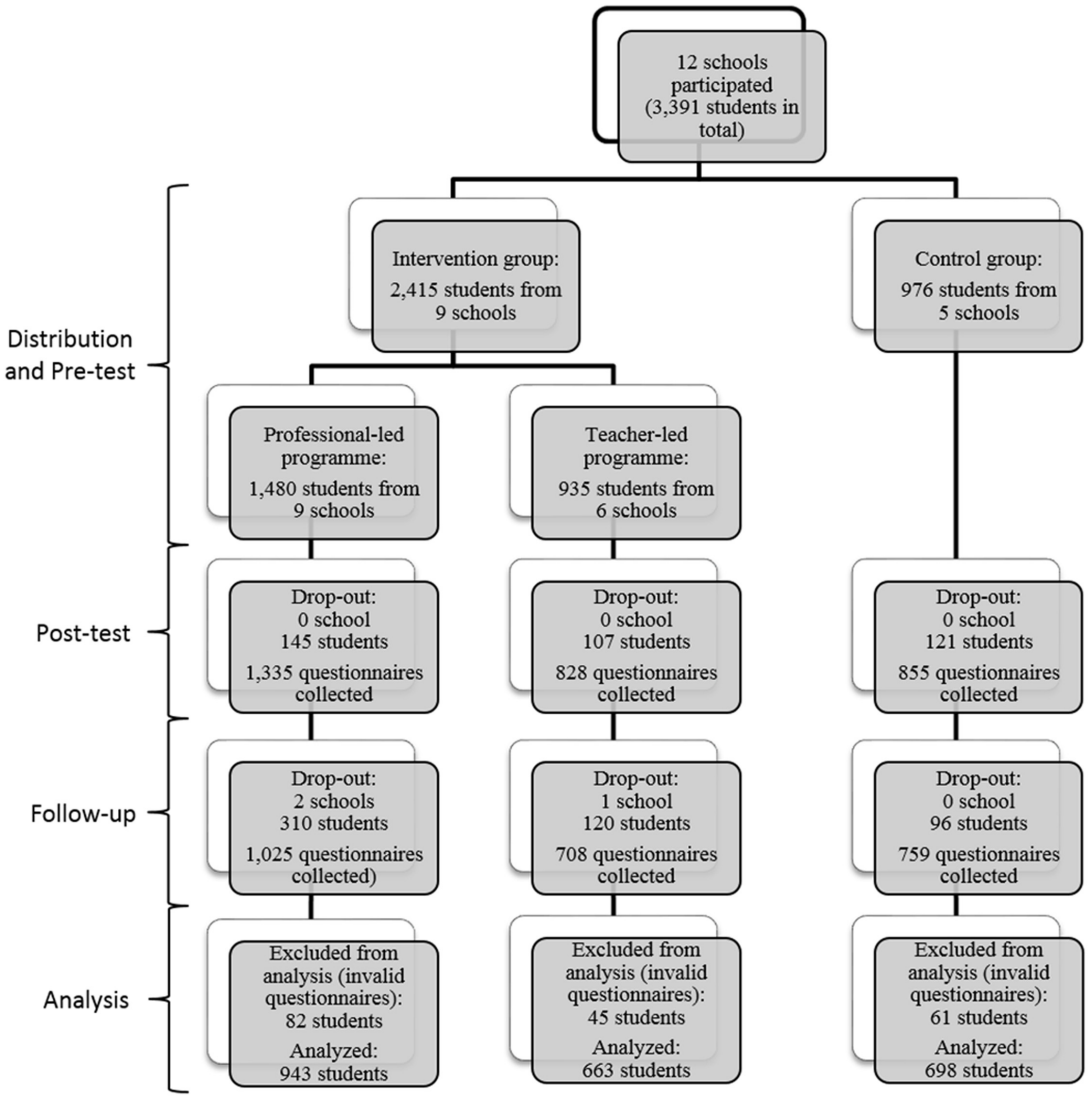
Flowchart of the study.

A total of 3,391 students participated in the study. Of the participating students, 66.4% were from Secondary 2, 28.7% were from Secondary 3, and 4.9% were from Secondary 4. Fig 1 shows the flowchart of participating students in the study. In total, 1,480 students participated in the professional-led programme, 935 in the teacher-led programme, and 976 in the control group. Post-test questionnaires were collected, numbering a total of 1,335 from the professional-led programme, 828 from the teacher-led programme, and 855 from the control group. The drop-out rates at post-test in these three groups were not significantly different statistically (9.8%, 11.4% and 12.4%, respectively; χ^2^ = 4.32, df = 2, p = 0.12). A follow-up evaluation was conducted 4 to 5 months after completion of the programme. Follow-up questionnaires were collected from the three groups, numbering 1,025, 708, and 759, respectively. The drop-out rates at follow-up (based on post-test) were 23.2%, 14.5%, and 11.2%, respectively, which did differ significantly (χ^2^ = 58.93, df = 2, p<0.01). Two professional-led schools and one teacher-led school entirely dropped out at follow-up evaluation, resulting in the relatively high drop-out rates. In addition, the drop-out rate of males was significantly higher than that of females for both post-test evaluation (13.7% vs. 7.1%; χ^2^ = 38.12, df = 1, p<0.01) and follow-up evaluation (18.8% vs. 15.8%; χ^2^ = 4.64, df = 1, p = 0.03). Questionnaires with missing entries of more than 20% (i.e. more than 12 missing questions) or sloppy completion (such as having the same response for all items in three measures of the questionnaire) were treated as invalid and excluded from the analysis.

In the end, 2,304 students remained for further analysis. The sample was roughly equally shared by female (n = 1,135; 49.4%) and male students (n = 1,162; 50.6%) (Table 1). The gender distribution across the three groups was statistically different (χ^2^ = 83.58, df = 2, p<0.01), with more female students enrolled in the professional-led group (53.5%) and in the teacher-led group (58.5%), but more male students enrolled in the control group (64.7%). The mean age of the students was 15.1, with standard deviation 1.0. Those enrolled in the teacher-led group were slightly younger (mean = 14.5; s.d. = 0.8) than those in the other two groups. The programme started at different times for different schools and groups (as reflected in the time of the pre-test assessment). Around 75% of students under analysis started the programme at the beginning of a school year (i.e. in September and October). There were more students participating in the professional-led group, who started the programme at the beginning of a school year.

**Table 1:** Basic information of students separated by professional-led group, teacher-led group, and control group.

	Professional-led	Teacher-led	Control	All groups
	n (% of total)	n (% of total)	n (% of total)	n (% of total)
**Gender**^1^	**938 (100)**	**662 (100)**	**697 (100)**	**2,297 (100)**
Female	502 (53.5)	387 (58.5)	246 (35.3)	1,135 (49.4)
Male	436 (46.5)	275 (41.5)	451 (64.7)	1,162 (50.6)
**Programme starting time**^2^	**941 (100)**	**663 (100)**	**698 (100)**	**2,302 (100)**
Sept / Oct	825 (87.7)	427 (64.4)	467 (66.9)	1,719 (74.7)
Otherwise	116 (12.3)	236 (35.6)	231 (33.1)	583 (25.3)
**DASS21—Depression**^3^	**923 (100)**	**646 (100)**	**666 (100)**	**2,235 (100)**
Score<12	710 (76.9)	485 (75.1)	467 (70.1)	1,662 (74.4)
Score≥12	213 (23.1)	161 (24.9)	199 (29.9)	573 (25.6)
**Age**^4^				
Mean	15.5	14.5	15.3	15.1
Standard deviation	1.0	0.8	1.0	1.0

^1^ Gender was missing for 7 students.

^2^ Programme starting time was missing for 2 students.

^3^ Depression score was missing for 69 students.

^4^ Age was missing for 12 students.

### Differences at pre-test assessment

Table 2 summarizes the scores of all outcome measures during pre-test, post-test, and follow-up assessments across the three groups. At pre-test assessment, the group effects were not significant for outcome measures except for help-seeking towards friends (F = 6.68, p<0.01) and the stress subscale of DASS21 (F = 3.47, p = 0.03). Students from the control group scored slightly lower in help-seeking behaviour towards friends, while those from the teacher-led group scored higher in stress symptoms. 80%, 65% and 84% of all three groups of students were either at the normal range or had mild symptoms of depression, anxiety and stress, respectively.

**Table 2:** Pre-test, post-test, and follow-up mean scores (standard deviation) by group, and test of intervention effects from multi-level modelling.

		Control			Professional-led					Teacher-led				
								*βcoefficient for interaction term*					*βcoefficient for interaction term*	
*Outcome Measure*	*Range*	*Pre-test*	*Post-test*	*Follow-up*	*Pre-test*	*Post-test*	*Follow-up*	T1*Prof	T2*Prof	*Pre-test*	*Post-test*	*Follow-up*	T1*Teach	T2*Teach
**Knowledge**	0–6	2.24	2.27	2.21	2.58	3.74	3.15	1.15*	0.60*	2.50	3.43	3.06	0.92*	0.59*
		(1.19)	(1.17)	(1.25)	(1.23)	(1.52)	(1.42)			(1.20)	(1.40)	(1.41)		
**DASS21**														
Depression	0–42	7.90	9.20	9.67	7.09	8.30	9.02	0.09	0.31	7.56	9.23	8.53	0.47	-0.71
		(8.29)	(9.27)	(9.29)	(7.75)	(8.37)	(8.94)			(7.67)	(9.09)	(8.50)		
Anxiety	0–42	8.40	9.26	9.60	7.45	8.42	8.59	0.17	-0.07	8.35	9.54	8.54	0.34	-1.02*
		(7.43)	(8.48)	(8.82)	(6.53)	(7.96)	(8.12)			(7.30)	(8.51)	(8.03)		
Stress	0–42	10.59	11.72	11.57	10.06	11.09	11.19	0.05	0.29	11.37	12.17	10.79	-0.20	-1.39*
		(8.83)	(9.30)	(9.29)	(8.10)	(9.08)	(8.88)			(8.34)	(9.05)	(8.77)		
**Attitude**	-12–12	1.28	1.39	0.97	1.65	2.24	2.22	0.50*	0.90*	1.67	2.13	2.08	0.38	0.75*
		(3.65)	(3.91)	(3.68)	(3.57)	(3.72)	(3.69)			(3.92)	(3.99)	(3.94)		
**MSPSS-C**														
Family	4–28	17.49	17.61	17.72	18.63	18.33	18.72	-0.39	-0.17	18.37	18.39	19.05	-0.09	0.49
		(6.96)	(6.61)	(6.17)	(6.53)	(6.77)	(5.89)			(6.75)	(6.76)	(6.15)		
Friend	8–56	36.93	37.01	36.81	39.09	38.64	39.07	-0.54	-0.01	39.84	39.10	39.64	-0.92	-0.16
		(13.19)	(12.81)	(12.06)	(11.85)	(12.30)	(10.89)			(12.14)	(12.49)	(11.75)		
**Help seeking**														
Professional	1–4	2.24	2.26	2.34	2.18	2.24	2.27	0.03	0.00	2.16	2.18	2.34	0.00	0.09
		(1.06)	(1.03)	(1.04)	(0.98)	(0.99)	(1.00)			(0.95)	(0.99)	(1.03)		
Family	1–4	2.74	2.74	2.78	2.87	2.82	2.83	-0.05	-0.09	2.89	2.82	2.90	-0.07	-0.03
		(1.07)	(1.04)	(1.00)	(1.02)	(1.01)	(0.98)			(1.04)	(1.03)	(0.98)		
Friend	1–4	3.11	3.15	3.15	3.32	3.26	3.30	-0.10*	-0.05	3.37	3.23	3.28	-0.17*	-0.11*
		(1.00)	(0.94)	(0.93)	(0.83)	(0.83)	(0.82)			(0.82)	(0.89)	(0.84)		
Teacher	1–4	2.33	2.36	2.44	2.25	2.32	2.35	0.05	0.00	2.22	2.24	2.44	0.00	0.12
		(1.05)	(1.03)	(1.03)	(0.98)	(1.00)	(1.00)			(0.97)	(1.00)	(1.03)		

* *p*<0.05

Multilevel modelling on pre-test assessment showed that six outcome measures—including all depression, anxiety, and stress subscales of DASS21, as well as help- seeking behaviour towards family and teachers—showed no significant differences between male and female students. Of those outcome measures with significant gender differences, female students scored better than male students in knowledge, attitude towards people with mental illness, and MSPSS.

For help-seeking behaviour when facing emotional distress, students’ scores showed a significant difference among the four choices (χ^2^ = 1588.93, p<0.01). Regardless of which groups participated, the patterns were the same. Students preferred to seek help from friends; then, ranked in order, sought help from families, teachers, and professionals.

### Intervention effect

**Fig 2:**
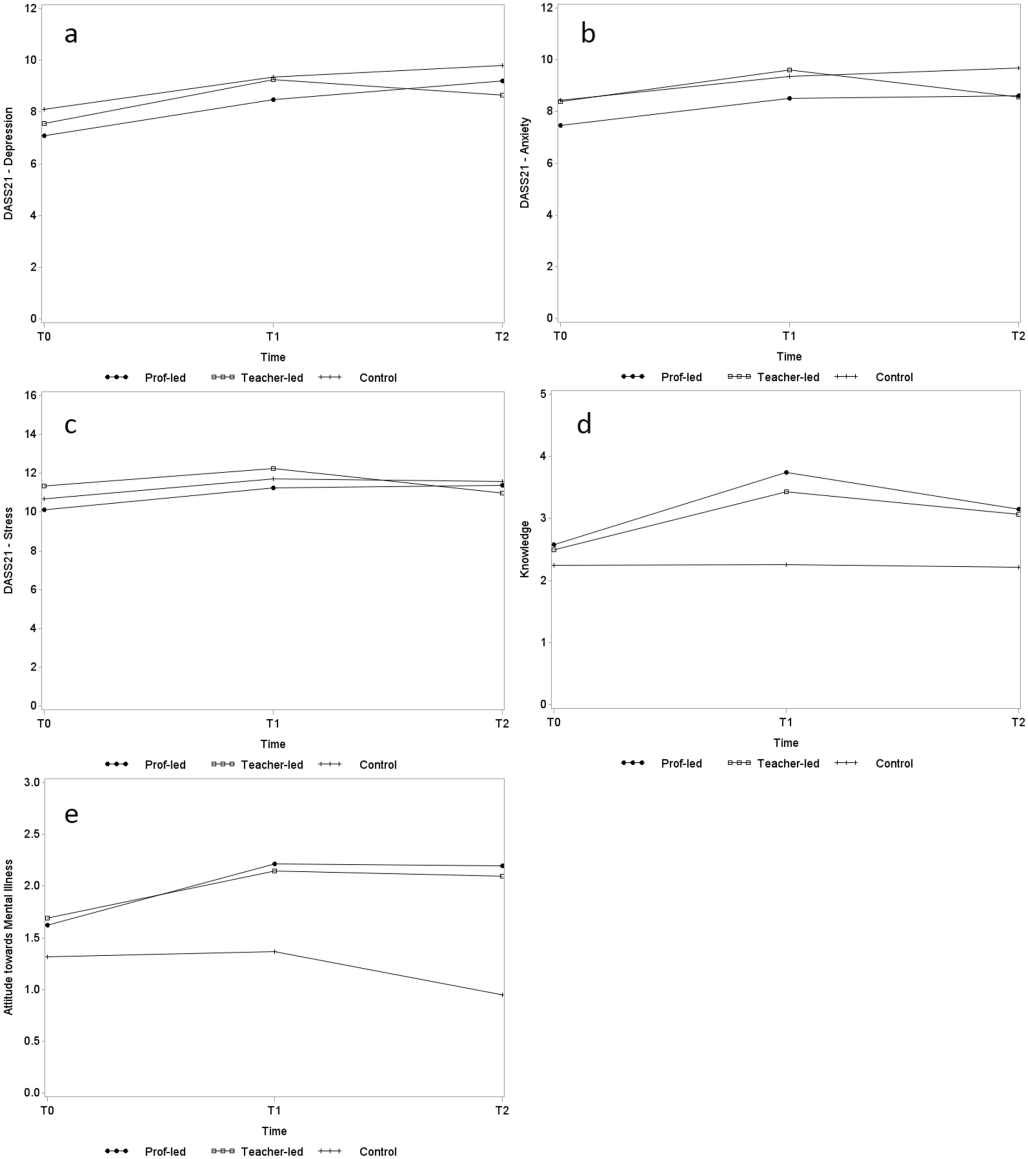
Interaction graphs of estimated change over time of primary and secondary outcomes (a-e). (a) Depression. (b) Anxiety. (c) Stress. (d) Knowledge. (e) Attitudes towards mental illness.

Table 2 also shows the results of estimated interaction effects from multi-level modelling for each outcome measure. Significant results were found for knowledge, DASS21, attitudes towards people with mental illness, and help-seeking behaviour towards friends. Fig 2 illustrates the intervention effects with the aid of interaction graphs. For the three conditions measured by DASS21, students generally experienced increases in scores during post-test and follow-up assessments when compared to pre-test, as shown by Fig 2a–2c. However, the significantly negative coefficients of interaction term T2*teacher-led indicated significant but delayed intervention effects in anxiety (*β* = -1.02, p = 0.02) and stress (*β* = -1.39, p<0.01) symptoms for students enrolled in the teacher-led programme. The two scores prominently reduced for students enrolled in the teacher-led programme from post-test to follow-up assessment, but remained at similar levels for those enrolled in both the professional-led programme and the control group. No significant intervention effect was observed in depression (*β* = -0.71, p = 0.12). A power analysis was conducted to determine the achieved statistical power for these significant results. The statistical power exceeded 89% for knowledge, stress symptoms and attitude towards mental illness, and was around 77% for anxiety symptoms.

Clinical significances of the intervention on anxiety and stress symptoms between post-test and follow-up assessments were then examined. The cutoff scores separating a normative sample and an outpatient sample presented in Ronk et al. [38] were used to classify the recovered students (6.31 for anxiety and 12.42 for stress). Results are shown in Table 3. No clinically significant change could be observed in 71% to 77% of students for the two symptoms across different conditions. Nonetheless, students enrolled in the teacher-led programme had higher rates of recovery but lower rates of deterioration.

**Table 3:** Rates (%) of recovered, improved, no change, and deteriorated between post-test and follow-up assessment for anxiety and stress symptoms, DASS21.

*Outcome measures*	*Recovered*	*Improved*	*No change*	*Deteriorated*
**Anxiety**				
Professional-led	7.1	4.6	74.6	13.7
Teacher-led	10.5	6.8	71.2	11.5
Control	7.3	6.4	72.1	14.2
**Stress**				
Professional-led	7.9	4.6	76.7	10.8
Teacher-led	9.1	4.8	76.4	9.7
Control	7.1	6.2	75.4	11.3

Remarks (adopted from the study by Ronk et al. [38]): *(a) recovered*, when a person has made a positive reliable change and moved into the normal range; *(b) improved*, when a person has made a positive reliable change without moving into an adjacent range; *(c) unchanged*, when a person has not made a reliable change in either direction; and *(d) deteriorated*, when a person has made a negative reliable change.

The intervention effects were most prominent in knowledge. Immediately after completion of the programme, knowledge of mental health significantly increased for students in the professional-led programme (*β* = 1.15, p<0.01) and in the teacher-led programme (*β* = 0.92, p<0.01). Attitudes towards people with mental illness also became more positive, but failed to draw statistical significance at the 0.05 level for the teacher-led group (*β* = 0.38, p = 0.07). Follow-up assessment showed that these intervention effects persisted, although the effects on knowledge moderately declined (professional-led: *β* = 0.60, p<0.01; teacher-led: *β* = 0.59, p<0.01). These are reflected in Fig 2d and 2e.

The willingness to seek help from friends reduced after the programme with statistical significance, but the effect was very little (professional-led: *β* = -0.10, p<0.05; teacher-led: *β* = -0.17, p<0.05). Help-seeking behaviour towards teachers slightly increased (*β* = 0.12) for students enrolled in the teacher-led programme during follow-up assessment, but was barely non-significant statistically (p = 0.053). No significant change was observed in other categories. Overall, students’ help-seeking behaviour did not alter, with friends still being their first choice and professionals their last. For perceived social support, no statistically significant change was detected in either the professional-led or teacher-led groups.

### Knowledge of, and attitudes towards, people with mental illness over time

**Fig 3:**
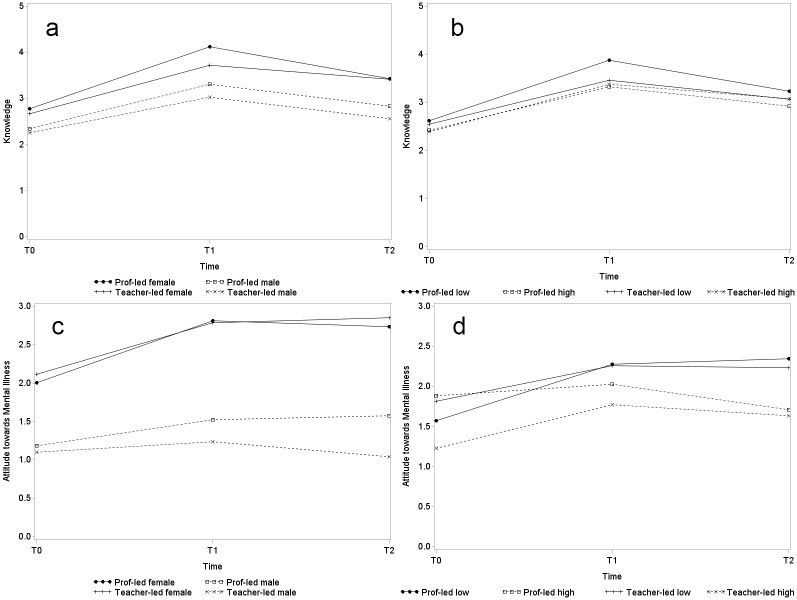
Interaction graphs of estimated change over time on knowledge and attitudes towards mental illness across genders and depression levels (a-d). **(a)** Knowledge across genders. (b) Knowledge across depression levels. (c) Attitudes across genders. (d) Attitudes across depression levels.

As reflected in the results of the follow-up assessment, the improvement in participants’ knowledge of mental health, and attitudes towards people with mental illness could still be maintained 4 to 5 months after intervention. Therefore, a three-way interaction model was applied to investigate if such continued effects differed across genders and across students with different levels of depressive symptoms. Students with initial scores (pre-test) of 12 or above on the depression subscale of DASS21 were categorized as a “high depressive symptom group”, as a 75^th^ percentile was found at 12. Table 1 also shows the number of students having this super-threshold score separated by group. Students enrolled in the control group were more likely to have higher initial depression scores (29.9% vs. 23.1% and 24.9% of the professional-led and the teacher-led groups, respectively). Regression results are presented as interaction graphs in Fig 3 (the control group is excluded from these figures). Improvement in knowledge suffered a slight reduction during follow-up assessment in both males and females (Fig 3a), and in low and high depressive symptom groups (Fig 3b). Such a reduction was not statistically different across gender in either the professional-led group (*β* = 0.04, p = 0.82) or the teacher-led group (*β* = -0.24, p = 0.20), but was statistically significantly different in students of the teacher-led group across different depression levels (*β* = 0.63, p<0.01). The knowledge of students with a relatively high depressive level in the teacher-led group could be maintained better than the low depressive group. Attitudes towards people with mental illness were always more positive among female than male students, and the positive change in attitude could be maintained by all at follow-up except for the male students in the teacher-led group (Fig 3c). In the professional-led group, those students classified with high depressive symptoms did not show significant improvement in attitudes during post-test assessment and even showed a slight reduction at follow-up, as reflected in Fig 3d. In general, students with a low depression level had better attitudes towards people with mental illness than did the high depressive symptom group.

## Discussion

Despite high levels of cooperation between the participating schools—including their teaching personnel—and the research team, we found no evidence that the LPD programme led by professionals reduced depression, anxiety, or stress symptoms at the post- and follow-up periods under diverse everyday school conditions. On the contrary, the level of depression, anxiety, and stress increased at post-test for students in both the intervention group and the control group. Meta-analysis studies [39] and systematic reviews [11] generally provide evidence that school-based prevention programmes do reduce depressive symptoms. However, we observed that this programme not only did not achieve prevention effects but that professional-led school-based programmes coincided with an increased number of negative outcomes both immediately and 4 to 5 months after the end of the programme. The possibility of a harmful effect is minimal, but it should be acknowledged that (1) the pattern may be a reflection of greater self-perception and sensitivity towards symptoms of depression, and (2) there was a “stress cycle” for our students as most of the important examinations were scheduled during the period of the study. Nevertheless, the possibility of negative outcomes after school-based programmes has also been found for other mental disorders, such as attention-deficit-hyperactivity disorder [40] and depression [41], and an improved understanding of the mechanisms underlying possible adverse outcomes merits further investigation.

Interestingly, although the symptoms of depression, anxiety, and stress increased among students of the professional-led and control groups at post-test, students of the teacher-led group showed decreased levels of depression, anxiety, and stress at the follow-up assessment, and their level of anxiety and stress displayed a statistically significant reduction. Although the effects were small, this finding seems to indicate that teachers could effectively deliver such school-based programmes, especially in reducing students’ anxiety and stress. In terms of clinical significance, results showed that more percentage of students in the teacher-led group either improved or recovered (made a positive reliable change in anxiety and stress symptoms) than the professional-led group, and professional-led group of students had higher percentage of deteriorating (that is, made a negative reliable change in anxiety and stress symptoms). Nevertheless, one should take note that the current sample was from general population, over 70% of the participants remain unchanged in their level of anxiety and stress, which was in a normal range. In addition, we suspect that the major causes of the students’ stress, anxiety, and depression are probably academically-related; having teachers as the trainers, therefore, enhances the practical application of the taught skills and concepts for the students as well as increasing the motivation of the students to learn those knowledge and skills. Most importantly, the trained teachers who taught this programme were able to provide continuous support to students whenever they felt distressed throughout the school year; the instructors of the professional-led group were unable to provide this kind of support. Similar findings concerning school staff as providers of universal prevention programmes for students’ anxiety and depression are reported in the meta-analysis conducted by Mychailyszyn et al. [39].

However, our findings were contrary to previous studies like Wahl et al. [17], which suggested that psychologists were more effective to deliver depression prevention programme in schools than schoolteachers, especially for female students. Their study showed that the depressive levels for girls were significantly lower than the teacher-led and control conditions at 12-month follow up. However, the two studies might not be directly comparable as the LPD programme was carried out in usual classroom setting, depending on the school type (either co-ed or boys or girls school) with shorter follow-up period (only 4–5 months) but the Wahl et al. study intentionally divided the students by gender and conducted the programme to gender homogenous groups to study the gender effects of their programme and observed the changes of students up to 12 months after the intervention. With the small effects of the current study, it was not possible to conclude whether schoolteachers were more effective to deliver mental health programme than mental health professionals, but this study provided new evidence to support teachers’ effectiveness in implementing such programmes. Thus, schoolteachers may be an alternative source of instructors instead of relying on mental health professionals like psychologist alone to conduct mental health programmes in schools, and each type of instructor has its own merits.

Besides the main outcomes about depressive, anxiety, and stress symptoms, we also explored the potential changes in secondary outcomes, especially those from a positive aspect; that is, knowledge of mental health and attitudes towards mental illness. We found that the knowledge of mental health was significantly higher in the intervention groups than in the control group at both post-test and the follow-up assessment. This demonstrated that the programme was effective in disseminating knowledge to the students, whether by trained mental-health professionals or by schoolteachers, and the knowledge could be sustained for 4 to 5 months. In addition, intervention group students held more positive attitudes towards people with mental illness than did the control group students. With less prejudice against mental illness, they may be more willing to seek help when they or their family and friends encounter mental health problems. However, the perceived social support and help-seeking attitude of students were stable in all three conditions (the professional-led, teacher-led and perceived social support) and did not have significant changes across time. Probably it is because perceived social support and help-seeking attitude are not easily changed and the LPD programme did not have direct effect on these two constructs, as it focused on different knowledge and skills to promote student mental health.

Additional analysis was conducted to explore the influence of potential confounding variables on the primary and secondary outcomes. We found that enhanced knowledge of mental health at post- and follow-up assessments was maintained regardless of gender and the kind of instructors. However, with the teacher-led condition, this continuous effect was better in the high depressive group than in the low depressive group. That means the LPD programme is able to enhance the knowledge of students of both genders, and the results supported the effectiveness of schoolteachers in delivering such mental-health programmes; it is important to note that the programme may be especially useful for students with higher levels of depressive symptoms.

In our study, we did not find a statistically significant difference between male and female students in the level of depressive, anxiety and stress symptoms at pre-test. This seems to be contradicting the epidemiological findings of gender difference in the prevalence of depression in adolescence, with adolescent girls more likely to develop depression than boys (about 2:1) [42]. However, our current study only assessed the depressive symptoms among a sample of adolescents mainly in Grades 8–10, with mean age of 15.1, in which gender difference was just about to emerge [43–44]. Some previous studies even suggested the gender difference began at Grade 12 [45]. In fact, our sample also showed higher level of depressive symptoms for female students aged 17 and 18. Therefore, our findings did not have high discrepancy with the results from epidemiological studies.

For this study, we had a larger sample size than in the pilot study; we compared the relative effectiveness of the professional-led and teacher-led conditions, which provided important information on whether trained schoolteachers could be as effective as mental health professionals in delivering a mental-health programme for adolescents. The results demonstrated that schoolteachers delivering the programme might achieve similar or even better outcomes than mental-health professionals in certain situations. The possible reasons include: (1) teachers are adept at teaching knowledge and skills; (2) they know their own students better than external instructors do, so they can provide more relevant examples to demonstrate the concepts and remind specific students to pay attention to specific skills, for example, students with high stress to practice more breathing exercises; and (3) they can reinforce the knowledge and skills through encounters with students that are not limited to the designated teaching hours of the programme. The inclusion of a follow-up assessment in the present study also revealed the important role of teachers in supporting students on mental-health or non-academic issues after completion of the programme; that is, in a normal school routine. Our findings in the follow-up assessment showed that there were reductions in students’ level of anxiety and stress in the teacher-led condition but not in the professional-led nor control conditions, which supported our speculation in the pilot study that teachers’ willingness to listen to students’ feelings and concerns may promote the positive wellbeing of students, and that the presence of teachers throughout the school year is an important source of support to students.

The review conducted by Han & Weiss [46] on sustainability of teacher implementation of school-based mental health programme also suggested some other reasons that might be relevant to our findings and LPD programme. They suggested the importance of the administrative support from the school principal, teacher’s beliefs in their teaching efficacy, the acceptability of the programme principles, programme effectiveness, adequate and quality teachers’ training, and ongoing support and sufficient resources were all important factors whether the programme can be sustained in school with implementation fidelity. In the LPD programme, we indeed received the support from the school principal to carry out the programme as well as the evaluation study in the participating schools. For those teachers who taught the programme in the teacher-led phase, most were the guidance teachers or had observed how the programme was delivered by the mental health professionals in the professional-led phase. They also attended a 3-hour training workshop before implementing the programme to enhance their understanding of the rationale, theoretical framework and effectiveness of the LPD programme. All teaching materials were also provided to each teacher. Therefore, it is believed that they were equipped with the knowledge and skills to deliver the programme on their own. Besides, the research team would pay visit to the school when the programme had started, to ensure the programme fidelity and provide support to teachers when necessary. All these factors might have contributed to the positive effects we detected for the teacher-led condition.

Thus, our findings suggest that schoolteachers may be valid instructors for the delivery of a school-based universal prevention programme for adolescent depression, such as the LPD programme in Hong Kong reported here. If school principals share the vision of this kind of mental-health programme, and are willing to implement it at their own schools by releasing teachers to receive proper training, the programme can probably be sustained in schools, to the long-term benefit of their students.

### Limitations and Future Direction

Our study has several limitations. First, the sample of this study was drawn from those schools which enrolled in the LPD programme and it is a quasi-experiment but not randomized controlled trial; the participating schools which completed both the professional-led and the teacher-led phases might have a higher readiness than other schools to implement this kind of school-based universal prevention programme of adolescent depression. Therefore, there might be self-selection bias and the results might not be generalizable to non-participating schools. In addition, the teaching schedule of participating schools varied, so that some students attended a lesson weekly for 12 weeks while some completed the programme in only 6 to 8 weeks, with double lessons each time—this might have adversely affected the latter group as these students needed to learn more than one topic each time and they might not have had enough time to understand thoroughly the topics being taught. The implementation period of the programme might also have affected the results. As two schools from the professional-led group started the programme in the second term of the school year, it would be difficult for the research team to conduct follow-up assessments: in the following school year, both teachers and students might have changed. This is in fact the reason for the high drop-out rate at follow-up for the professional-led group, which reduced the sample size for analysis.

The present study was a larger-scale study than that of our previous pilot study. The sample size had expanded and had a lower attrition rate; however, no significant reduction in depression scores was observed. As explained above, it is difficult to detect changes of symptomatology in the participants of a universal prevention programme as most of them are not at risk. In order to assess the effectiveness of a universal programme in future studies, it would be more informative to include structured clinical interviews for the assessment of depression among students, and compare the number of new cases of depression between the intervention group and the control group during the follow-up period, instead of measuring the level of depressive symptoms alone [14]. The onset of depression or depressive episodes may inform us about the preventive effect of the programme. The follow-up period could also be extended to a longer time (e.g. 6–12 months) to explore the longer-term effects of the LPD programme as it was suggested that the intervention was taking place from baseline to post-intervention, any intervention effect might only manifest after the intervention [17]. From a recent meta-analysis conducted by Stice and colleagues on depression prevention programmes, they found that at least 6 months were needed for positive outcomes to be achieved [47]. Measures, then, should be taken to encourage higher participation in follow-up assessments in months or even years following completion of the programme.

The help-seeking attitude of students when under emotional distress was noteworthy. Regardless of whether the students were from the intervention groups or the control group, our results showed that students preferred to seek help first from friends, family, and teachers, and only then professionals. Therefore, future universal prevention programmes should involve parents or peers as well, who should be given guidance on how to provide emotional support to their child or friends with emotional distress, enhance their understanding of the importance of seeking help from mental-health professionals when necessary and how to connect their child or friends to available resources.

## Conclusion

The present study evaluated the effectiveness of a school-based universal prevention programme for adolescent depression in Hong Kong. Results showed that this programme was effective in enhancing the knowledge of students about depression and stress-management skills, as well as in improving students’ attitudes towards mental illness, irrespective of whether the instructors were mental-health professionals or schoolteachers. Although the programme might not have reduced the depressive symptoms of the intervention group students, students’ levels of anxiety and stress reduced significantly a few months later when the programme was conducted by schoolteachers. These findings support that schoolteachers may be effective in delivering school-based universal prevention programmes for youth depression or mental health in general. Yet, the effects found in the present study are small so more research is needed to understand how effective schoolteachers are in implementing mental health programmes. Sustainability of this kind of school-based programme is more likely to happen if some important conditions can be fulfilled: (1) support from school principals; (2) perceived relevance by teachers and students; (3) appropriate and quality training is given to teachers, (4) willingness of teachers to learn and implement this programme with space and spare teaching hours; (5) programme flexibility; and (6) availability of continuously updated teaching materials and consultation support from the research team.
